# Sexual Quality of Life in Postmenopausal Women: A Comparative Randomized Controlled Trial of Intravaginal PRP Therapy Versus Local Hormonal Treatments

**DOI:** 10.3390/medicina61071140

**Published:** 2025-06-25

**Authors:** Geanina Sacarin, Ahmed Abu-Awwad, Nitu Razvan, Marius Craina, Bogdan Hogea, Bogdan Sorop, Simona-Alina Abu-Awwad, Mircea Diaconu, Nicolae Ciprian Pilut, Madalina-Ianca Suba

**Affiliations:** 1 Doctoral School, “Victor Babes” University of Medicine and Pharmacy, 300041 Timisoara, Romania; geanina.sacarin@umft.ro (G.S.); madalina.suba@umft.ro (M.-I.S.); 2 Department XV—Discipline of Orthopedics—Traumatology, “Victor Babes” University of Medicine and Pharmacy, 300041 Timisoara, Romania; ahm.abuawwad@umft.ro (A.A.-A.); hogea.bogdan@umft.ro (B.H.); 3 Research Center University Professor Doctor Teodor Șora, “Victor Babes” University of Medicine and Pharmacy, 300041 Timisoara, Romania; 4 Clinic of Obstetrics and Gynecology, “Pius Brinzeu” County Clinical Emergency Hospital, 300723 Timisoara, Romania; craina.marius@umft.ro (M.C.); bogdan.sorop@umft.ro (B.S.); diaconu.mircea@umft.ro (M.D.); 5 Department of Obstetrics and Gynecology, Faculty of Medicine, “Victor Babes” University of Medicine and Pharmacy, 300041 Timisoara, Romania; 6 Department of Microbiology, “Victor Babes” University of Medicine and Pharmacy, 300041 Timisoara, Romania; pilut.nicolae@umft.ro; 7 Dr. Victor Babes, Infectious Diseases and Pneumononcology Hospital Timisoara, 300310 Timisoara, Romania

**Keywords:** postmenopause, PRP, sexual dysfunction, vaginal health, hormone-free therapy, female sexual function index

## Abstract

*Background and Objectives*: Genitourinary syndrome of menopause (GSM) is a prevalent and distressing condition in postmenopausal women, often leading to sexual dysfunction characterized by vaginal dryness, pain, and reduced libido. While local estrogen therapy remains the standard treatment, due to safety concerns and contraindications, there is growing interest in the exploration of alternative interventions. This study aimed to compare the effectiveness and safety of intravaginal platelet-rich plasma (PRP) therapy versus local hormonal treatment in improving sexual function and vaginal health in postmenopausal women. *Materials and Methods*: A prospective, controlled clinical trial was conducted between January 2023 and December 2024 across three private gynecology clinics in Timișoara, Romania. Ninety postmenopausal women aged between 50 and 65 years with FSFI scores ≤ 23 were randomized into two groups: one receiving three monthly sessions of autologous PRP and the other undergoing 12 weeks of vaginal estriol therapy. Outcomes were assessed using validated tools—the Female Sexual Function Index (FSFI), the Vaginal Health Index (VHI), the Patient Global Impression of Improvement (PGI-I), and patient satisfaction scores—at baseline, week 6, and week 12. *Results*: Both of the treatment groups demonstrated significant improvements in FSFI and VHI scores at 12 weeks, with the PRP group showing a slightly higher, though not statistically significant, mean increase in the total FSFI (+10.1 vs. +9.3 points). Clinical gains were also observed in lubrication, elasticity, and dyspareunia. Patient satisfaction was high in both groups (93.3% PRP vs. 88.9% hormonal), and there were no reports of serious adverse events during the study period. The PRP group exhibited fewer side effects, without systemic symptoms, supporting its favorable safety profile. *Conclusions*: PRP therapy is a well-tolerated, hormone-free treatment that offers clinically meaningful improvements in sexual function and vaginal health, comparable to estrogen therapy. It may be particularly beneficial for women with contraindications to hormones or in advanced postmenopause. Further long-term studies are needed to confirm these findings and optimize treatment protocols.

## 1. Introduction

The menopausal transition represents a major physiological milestone in a woman’s life, defined by the cessation of menstruation and reduced ovarian estrogen output [1,2]. This endocrinological shift is associated with a wide range of physiological and psychological changes that may profoundly affect a woman’s overall quality of life [3,4]. Among these, sexual health disturbances are frequently reported and include symptoms such as vaginal dryness, dyspareunia, reduced libido, and diminished sexual satisfaction. These symptoms collectively contribute to the broader concept of a decreased sexual quality of life in postmenopausal women [5,6].

Sexual wellbeing is a multifactorial construct that encompasses physical, emotional, psychological, and relational dimensions. In postmenopausal women, sexual dysfunction is often a result of the genitourinary syndrome of menopause (GSM), formerly known as vulvovaginal atrophy. GSM involves structural and functional changes in the vaginal tissue, including epithelial thinning, diminished elasticity, poor blood flow, and reduced natural lubrication, all of which contribute to discomfort during sexual activity and lower sexual responsiveness [7,8,9,10,11]. Given the increasing recognition of sexual wellbeing as an integral component of overall health in women, addressing these concerns has become a clinical priority in menopausal care.

Conventional treatment approaches for GSM and related sexual dysfunctions have centered around hormonal replacement therapy (HRT), primarily involving local or systemic estrogen administration. Hormone therapy has demonstrated efficacy in restoring the vaginal structure, increasing moisture, and reducing symptoms such as discomfort during intercourse [12,13,14]. However, its use is often limited by contraindications, potential side effects, and declining patient acceptance due to long-term safety concerns, particularly related to cardiovascular risks and hormone-sensitive malignancies [15,16].

In recent years, regenerative medicine has emerged as a promising field for addressing sexual dysfunction in postmenopausal women. One such intervention is the intravaginal administration of platelet-rich plasma (PRP), a biologically active autologous product derived from centrifuged whole blood. PRP contains a high concentration of bioactive molecules, including PDGF, TGF-β, and VEGF, which are involved in tissue regeneration and angiogenesis. These molecules play critical roles in tissue repair, angiogenesis, and collagen synthesis [17,18]. PRP promotes local tissue renewal and improved blood flow in the vaginal mucosa, potentially enhancing vaginal health and sexual response without relying on hormone supplementation [19,20,21].

Despite the increasing interest in PRP as a novel therapeutic modality, clinical data regarding its efficacy and safety in the context of postmenopausal sexual dysfunction remain limited. Furthermore, direct comparisons between PRP and established hormonal treatments are scarce in the literature. As a result, clinicians lack evidence-based guidelines to inform the selection of optimal therapeutic strategies for this patient population.

The present study aims to address this gap by evaluating and comparing the effects of intravaginal PRP therapy and hormonal treatments on the sexual quality of life in postmenopausal women. By employing validated assessment tools and a structured clinical protocol, this investigation seeks to provide a comprehensive analysis of treatment outcomes, with a focus on patient-reported experiences and quality-of-life indicators. The findings of this study are intended to contribute to the growing body of evidence supporting individualized and effective approaches to sexual health management in the postmenopausal population.

**Figure 1:**
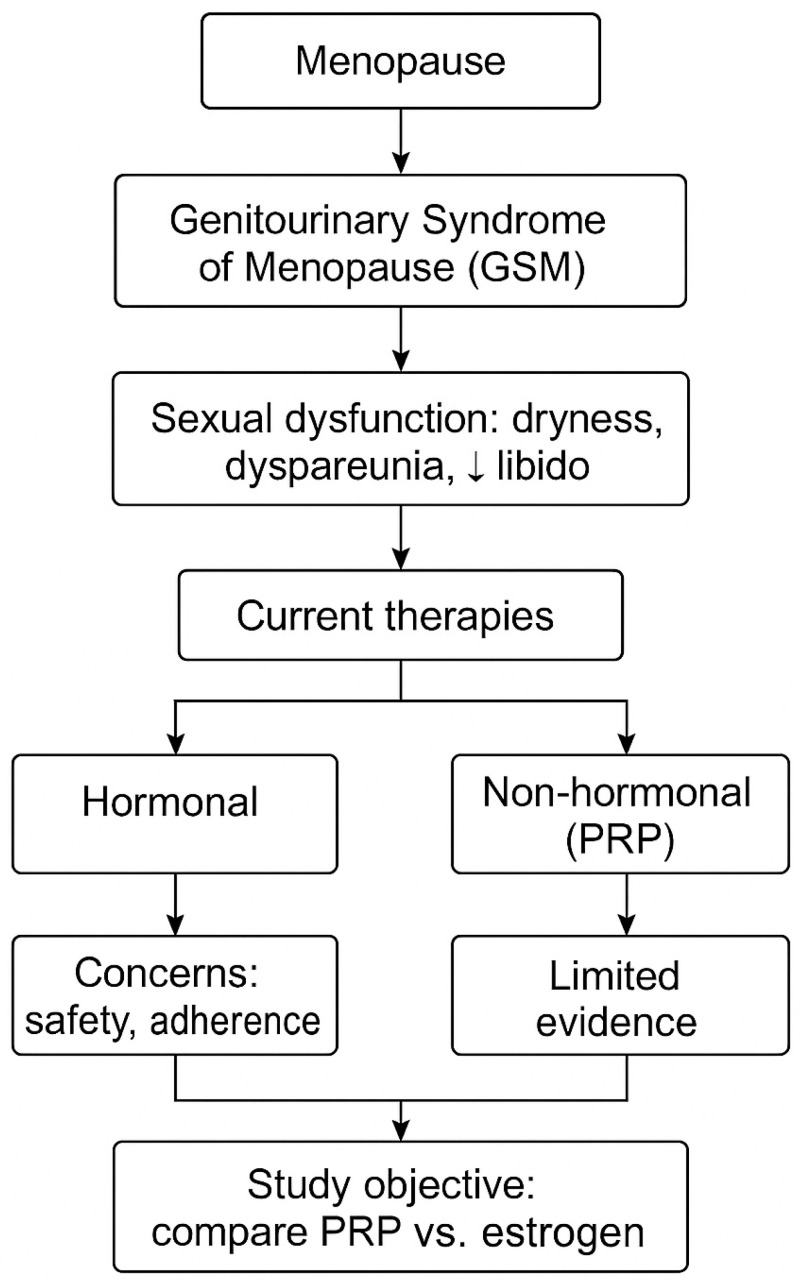
The conceptual framework of the study. ↓ indicates reduced.

A visual representation of the rationale behind the study design is presented in Figure 1, highlighting the cascade from menopause to GSM, its impact on sexual function, the limitations of current therapeutic options (hormonal and non-hormonal), and the rationale for comparing intravaginal PRP with local estrogen therapy.

## 2. Materials and Methods

### 2.1. Study Design

This prospective, controlled, comparative clinical study was conducted between January 2023 and December 2024 in Timișoara, Romania. The study was implemented across three private healthcare settings: one fully accredited gynecology clinic and two outpatient medical offices operating under a standardized clinical governance protocol.

The primary aim of the study was to evaluate the efficacy, safety, and tolerability of intravaginal PRP therapy as compared to standard local estrogen therapy in improving the sexual quality of life and vaginal health of postmenopausal women. The secondary aim was to explore whether PRP, as a regenerative, non-hormonal alternative, could offer comparable clinical outcomes without the systemic side effects commonly associated with estrogen exposure.

All the procedures conformed to the ethical standards described in the 2013 update of the Declaration of Helsinki. Ethical approval was obtained from the Independent Ethics Committee of the hosting institution (Approval no. 3/3 December 2022). Written informed consent was obtained from all the participants. The study design incorporated close participant monitoring, the standardized collection of patient-reported outcomes, and comprehensive safety documentation. No changes were made to the methods after trial commencement.

The trial was completed as originally planned; no interim analyses or early stopping criteria were applied.

### 2.2. Participants

The participants eligible for inclusion in the study were postmenopausal women aged between 50 and 65 years with confirmed natural menopause (≥12 months amenorrhea and FSH > 30 IU/L) [1]; the presence of moderate-to-severe symptoms of GSM, such as vaginal dryness, pain during intercourse (dyspareunia), reduced sexual desire, or general sexual discomfort; a total FSFI (Female Sexual Function Index) score of 23 or below, indicating clinically significant sexual dysfunction [22]; agreement not to begin any new hormonal, herbal, or local vaginal treatments throughout the study period; and the ability to give informed consent and fully participate in all study-related procedures.

Women were excluded from the study if they had a history of hormone-sensitive cancers (such as breast, ovarian, or endometrial cancer), ongoing or recurrent urogenital infections (including bacterial vaginosis, Candida, or herpes), or if they had used systemic or local hormonal treatments within the previous 6 months. Additional exclusion criteria included having undergone intravaginal PRP, laser, or radiofrequency therapies in the past year; known blood disorders affecting platelet function or coagulation; the current use of anticoagulants; active autoimmune diseases; poorly controlled diabetes (HbA1c greater than 8.5%); or any other serious medical condition deemed by the investigator to interfere with study outcomes. Women were also excluded if they had advanced pelvic organ prolapse (stage III–IV), vaginal stenosis, recent vaginal surgery (within the past 6 months), psychiatric conditions that could affect sexual function or informed consent, or if they had participated in another interventional study within 30 days before enrollment.

To confirm eligibility, all the participants underwent a physical examination, a review of their medical history, a transvaginal ultrasound, and laboratory testing, including FSH, estradiol, and a complete blood count.

### 2.3. Interventions

The participants in the PRPG received three intravaginal sessions of autologous PRP, with four-week intervals between them (weeks 0, 4, and 8). The PRP was prepared in sterile conditions using a two-step centrifugation method, which allowed for obtaining a platelet concentration 4–5 times higher than the baseline value.

For each session, 20 mL of peripheral venous blood was collected into a citrated tube, followed by a first centrifugation at 1500 rpm for 10 min (soft spin), and then a second centrifugation at 3200 rpm for 10 min (hard spin). After activation, 5 mL of PRP was injected into the anterior and lateral vaginal walls, using a 22G blunt-tip cannula, under sterile conditions and with the application of a local anesthetic gel.

To reduce variability and ensure the procedure was consistent, all the injections were performed by the same experienced gynecologist.

The participants in the HG followed a treatment consisting of vaginal ovules with estriol (0.5 mg), applied twice a week for 12 consecutive weeks.

Treatment compliance was checked using medication diaries filled out by the patients and by counting the unused ovules returned at the follow-up visits. During the treatment period, using any local lubricants or vaginal moisturizers was not allowed.

All the participants, regardless of their group, were instructed to avoid vaginal intercourse or inserting any foreign object into the vagina for 48 h after each treatment session.

### 2.4. Outcome

This study primarily aimed to measure the variation in FSFI total scores between baseline and 12 weeks post-treatment. The FSFI, a clinically validated, 19-item tool, evaluates six dimensions of female sexual health: sexual desire, arousal, lubrication, orgasm, satisfaction, and pain perception. The total score can range between 2 and 36, and a score below 26.55 is considered to indicate sexual dysfunction [23].

In addition to the main goal, the study also looked at several secondary outcomes. One of them was the Vaginal Health Index (VHI), which evaluates vaginal moisture, elasticity, pH, epithelial integrity, and secretion volume, with a total score between 5 and 25 [24]. Another tool used was the Patient Global Impression of Improvement (PGI-I), a 7-point scale ranging from “very much improved” to “very much worse,” to assess how the patients perceived the treatment’s effectiveness [25].

Satisfaction levels were assessed through a 5-point Likert scale ranging from complete dissatisfaction to high satisfaction. At the same time, any side effects were monitored, recorded both through structured interviews and spontaneous patient reports, and classified according to the Common Terminology Criteria for Adverse Events (CTCAE v5.0) [26,27].

All the evaluations were done at three key time points: at the beginning of the study (week 0), in the middle of the treatment (week 6), and at the end of the monitoring period (week 12). To reduce the risk of bias, the clinical staff who administered and recorded the questionnaires did not know which treatment group each patient was assigned to.

There were no changes to the pre-specified outcomes after trial commencement.

### 2.5. Sample Size and Randomization

The enrollment of 90 participants was determined through an a priori power analysis designed to detect a 4-point, clinically meaningful difference in the FSFI outcomes, assuming a standard deviation of 5.5, a power of 80%, and a significance level of α = 0.05.The participants were randomly assigned using a computer-based block randomization approach with variable block lengths to ensure balanced allocation (4–6) and stratified by their age group, the duration since menopause onset, and their baseline FSFI score (≤16 vs. 17–23). Allocation concealment was ensured through the use of opaque, sealed, and sequentially numbered envelopes prepared by an independent researcher not involved in the data collection or analysis.

### 2.6. Statistical Analysis

All the statistical analyses were conducted using GraphPad Prism version 6. Continuous variables, including the total scores of the FSFI and the VHI, were presented as the mean ± SD. Between-group comparisons for these outcomes were performed using the unpaired two-tailed Student’s t-test, assuming approximately normal distributions.

Categorical variables, such as responses to PGI-I, levels of patient satisfaction, and the occurrence of adverse events, were expressed as absolute frequencies and percentages. Comparisons between groups were carried out using the Chi-square test, while the Fisher’s exact test was applied in cases where the expected cell counts were below 5.

In addition, exploratory subgroup analyses were conducted based on age (≥60 years), the time since menopause onset (>8 years), and the BMI (≥30), using descriptive comparisons. These analyses were not pre-specified, were not adjusted for multiple testing, and were intended solely to identify potential clinical trends.

A two-sided *p* value of less than 0.05 was considered to indicate statistical significance. The required sample size was determined a priori based on a power analysis designed to detect a minimum clinically important difference of 4 points in the FSFI score, assuming an SD of 5.5, a statistical power of 80%, and a significance level (α) of 0.05.

## 3. Results

### 3.1. Baseline Characteristics

**Figure 2:**
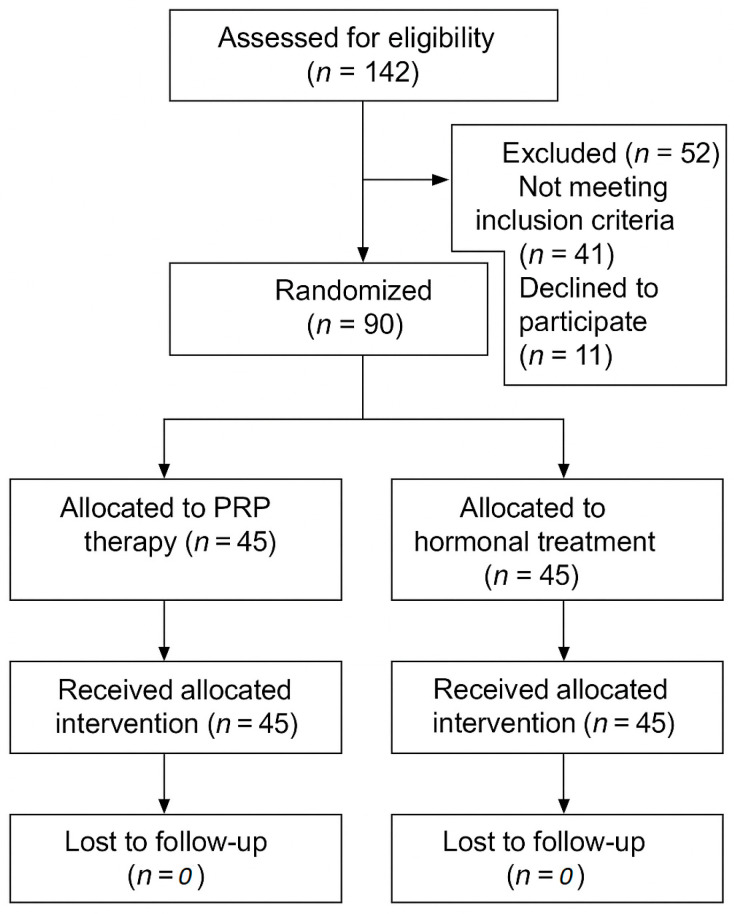
CONSORT flow diagram of participant progress through the study.

The participant flow through the study is illustrated in Figure 2, detailing the number of individuals assessed for eligibility, reasons for exclusion, randomization, allocation to treatment groups, and completion of follow-up. A total of 90 postmenopausal women were enrolled and randomized equally into the two treatment arms. All the participants completed the study protocol without dropping out. The trial was completed as originally planned; no interim analyses or early stopping criteria were applied.

As shown in Table 1, the two treatment groups were well matched across all the key baseline characteristics. There were no statistically significant differences between the PRP and hormonal groups in terms of the participants’ age, years since menopause, body mass index, marital status, smoking behavior, or prior use of local hormonal therapy (all *p* > 0.05). Importantly, the baseline values for both the FSFI and the VHI were comparable between the groups, ensuring that any subsequent differences in outcomes could be attributed to the interventions themselves, rather than initial disparities.

This homogeneity supports the internal validity of the study and enhances the interpretability of the comparative treatment outcomes. The absence of significant baseline imbalances strengthens the reliability of the observed treatment effects and suggests that randomization was successful in creating equivalent cohorts for the analysis.

### 3.2. Primary Outcome—FSFI

At 12-weeks post-intervention, both groups showed notable improvements in the total FSFI scores (Table 2). The women in the PRP group experienced a mean increase of 10.1 ± 3.4 points, reaching a post-treatment average of 28.3 ± 4.1. Similarly, the hormonal group showed a mean improvement of 9.3 ± 3.7 points, with a final average score of 27.9 ± 4.6. Although the difference between the two groups was not statistically significant (*p* = 0.19), the PRP group exhibited a trend toward greater improvement, suggesting a potentially more pronounced benefit to their sexual quality of life.

### 3.3. Secondary Outcomes

At 12 weeks, improvements in vaginal health and patient-reported outcomes were observed in both groups, with generally favorable trends for the PRP-treated women (Table 3).

VHI: In the PRP group, the VHI score increased from a baseline average of 12.3 ± 2.8 to 18.5 ± 3.2, reflecting a mean improvement of 6.2 ± 2.1 points. The hormonal group showed a comparable enhancement, with scores rising from 12.5 ± 3.1 to 18.1 ± 3.0, corresponding to a mean gain of 5.6 ± 2.3 points. While both groups experienced substantial benefits, their difference was not statistically significant (*p* = 0.14).

PGI-I: Most of the participants in both groups perceived a clear improvement. In the PRP group, 86.7% of women rated their condition as “very much improved” or “much improved”, compared to 82.2% in the hormonal group. This difference was not statistically significant (*p* = 0.52). Only a small fraction reported “minimal improvement”—4.4% in the PRP group and 6.6% in the hormonal group—without significant variation between the groups.

Patient Satisfaction (5-point Likert scale): Satisfaction levels were generally high across both interventions. In the PRP group, 64.4% of the patients reported being “very satisfied,” and 28.9% were “satisfied.” By comparison, 57.8% of the hormonal group reported being “very satisfied,” and 31.1% were “satisfied.” Neutral or dissatisfied responses were infrequent, noted in only 6.7% of the PRP-treated and 11.1% of the hormonally treated participants. Overall, combined satisfaction (satisfied + delighted) reached 93.3% in the PRP group versus 88.9% in the hormonal group, with no statistically significant difference (*p* = 0.37).

### 3.4. Adverse Events and Safety Profile

As summarized in Table 4, no serious adverse events occurred in either group, confirming the overall safety of both interventions. However, the PRP group exhibited a more favorable safety profile, with fewer adverse events and no systemic symptoms. All the PRP-related events were mild and transient, resolving spontaneously within 24–48 h without requiring intervention. In contrast, the hormonal group reported systemic side effects such as breast tenderness (8.9%) and vaginal spotting (4.4%), with one patient electing to discontinue treatment. While the differences were not statistically significant, the data underscore the tolerability of PRP therapy and support its use, particularly in patients with contraindications to hormonal therapy.

### 3.5. Subgroup Observations and Trends

As illustrated in Table 5, exploratory subgroup analyses revealed favorable trends for PRP therapy in several patient strata, although these were not powered for statistical comparison. Among the women aged ≥60 years, PRP led to greater improvements in the FSFI and VHI scores, particularly in the pain and lubrication domains. Similarly, in the women for whom >8 years had passed since menopause, PRP appeared to induce more sustained gains in vaginal health, while the hormonal group plateaued by week 6. In the small subgroup of women with a BMI ≥ 30, satisfaction levels were also higher with PRP. These trends suggest that PRP may offer incremental benefits in select subpopulations, potentially due to its regenerative mechanism of action, which may be independent of estrogen receptor sensitivity. No adjustment for multiple comparisons was performed as the secondary and subgroup analyses were exploratory in nature.

## 4. Discussion

This prospective comparative study evaluated the effects of intravaginal PRP therapy compared to local estrogen treatment on sexual function and vaginal health in postmenopausal women. The results showed that both therapies significantly improved the women’s sexual quality of life, as measured by FSFI and VHI scores, with no statistically significant differences between the two groups. Interestingly, PRP therapy resulted in slightly higher—but not statistically significant—improvements in several domains, including lubrication, pain reduction, and overall satisfaction, while also showing a more favorable safety profile.

In postmenopausal women, sexual dysfunction frequently results from estrogen depletion and the resulting deterioration of the urogenital tissue’s integrity and function. The decline in circulating estrogen leads to the thinning of the vaginal epithelium, a reduction in glycogen production, and the disruption of the local microbiota, resulting in an increased vaginal pH, epithelial fragility, and decreased lubrication—core components of the GSM. These changes impair not only sexual function, but also local immune defense mechanisms, increasing susceptibility to infections and mechanical trauma during intercourse [28,29].

Estrogen therapy has long been considered the gold standard for managing GSM-related symptoms. By binding to the estrogen receptors (ER-α and ER-β) present in vaginal and vulvar tissues, estrogen stimulates basal cell proliferation, the restoration of collagen and elastin fibers, and revascularization, leading to improved tissue elasticity and hydration. However, concerns remain about its long-term safety, particularly for women with contraindications, such as a breast cancer history or a thromboembolic risk. Moreover, in women who are more than 5–10 years postmenopause, the density of estrogen receptors may be significantly diminished, reducing the therapeutic efficacy of hormonal treatments [10,14].

In this context, PRP offers a hormone-free alternative that uses the body’s natural regenerative capacity through a concentrated mixture of autologous growth factors, such as PDGF, TGF-β, and VEGF. These factors act synergistically to promote fibroblast activation, collagen type III deposition, angiogenesis, and re-epithelialization, directly addressing the tissue degeneration seen in GSM without relying on hormone receptors [30].

The improvement in the FSFI scores observed in both groups aligns with previous research. Studies have shown that local estrogen therapy can increase sexual desire, vaginal lubrication, and overall satisfaction in postmenopausal women [31]. Similarly, other recent studies have found that intravaginal PRP can lead to the thickening of the stratified squamous epithelium, enhanced microvascular perfusion, and the reorganization of the extracellular matrix architecture [32]. One study reported significant improvements in VHI scores and vaginal atrophy indicators after three monthly PRP sessions, with the effects lasting up to six months [33].

A key advantage of PRP observed in this study was its excellent tolerability. No systemic adverse effects were reported in the PRP group, while the hormonal group experienced minor issues, such as breast tenderness and light vaginal bleeding. Similar findings have been reported by other studies, where over 90% of patients had no post-injection side effects [20]. These results support the idea that PRP, as an autologous and drug-free product, reduces the risk of systemic complications [34]. This is especially relevant for women with absolute or relative contraindications to hormone therapy, who often have limited treatment options.

Subgroup analyses suggested that PRP might be particularly beneficial for women aged ≥60 and those in later stages of postmenopause, where estrogen receptor expression is physiologically reduced and the local tissue response to estrogen is blunted. The continued increase in the VHI scores in these subgroups may indicate a delayed but progressive biological regeneration of the vaginal mucosa, a process dependent on tissue remodeling, rather than receptor activation. This observation aligns with findings by Salvatore et al., who reported the reduced effectiveness of local estrogen therapy in women beyond five years of menopause [21,35].

Although the differences between the PRP and the hormonal therapy groups were not statistically significant, the clinical improvements seen in the PRP group are notable. Parameters such as vaginal lubrication, tissue elasticity, and reduced dyspareunia showed meaningful clinical gains. These findings are consistent with the existing literature that highlights the potential of PRP in improving symptoms of female sexual dysfunction and the GSM [20,36].

This study has several strengths that support its methodological quality and clinical relevance. The prospective, controlled design with parallel groups and clear inclusion and exclusion criteria allowed for the recruitment of a well-defined and homogeneous postmenopausal population. The use of validated tools, such as the FSFI, VHI, and PGI-I, ensured reliable and reproducible assessments. Intervention protocols were standardized, and the PRP was prepared and administered by the same experienced clinician, minimizing procedural variability. Outcome assessments were performed by blinded evaluators, reducing any observer bias. Stratified randomization further improved the group comparability and the internal validity. Patient-reported outcomes, such as satisfaction and perceived improvement, were prioritized, which are especially important in studies addressing sexual function.

It is also worth noting that this is one of the few studies directly comparing autologous PRP therapy with standard hormonal treatment, providing valuable clinical data to guide therapeutic choices for women who cannot or prefer not to undergo hormonal treatment.

Despite these strengths, several limitations must be acknowledged. The study was conducted in a single metropolitan area, which may limit the generalizability of the results to populations with different sociodemographic or healthcare characteristics. Although the sample size was sufficient to detect clinically meaningful differences in the FSFI scores, it may not have been large enough to detect smaller, but still relevant, differences in secondary outcomes or subgroups.

These limitations are primarily explained by logistical and ethical considerations. For example, the short follow-up period (12 weeks) was selected to ensure patient adherence and limit dropout in this initial phase of investigation, while still capturing meaningful early clinical changes. Longer-term outcomes remain a priority for future studies.

Additionally, histological or molecular validation was not feasible in this population due to the invasiveness of vaginal biopsies and the associated ethical implications, especially in asymptomatic participants. Nonetheless, clinical and patient-reported outcomes provide substantial value in the context of symptom management and the quality of life.

Another limitation, the lack of participant blinding, was inherent to the nature of the interventions. However, the use of blinded outcome assessors and validated instruments minimized the risk of any significant bias.

Based on the present findings, intravaginal PRP can be considered a safe and potentially effective non-hormonal option for the management of GSM-related sexual dysfunction in postmenopausal women. Its favorable safety profile and patient-reported benefits make it a viable alternative for those with contraindications to hormone therapy or those who decline hormonal treatment.

In clinical settings, PRP may be cautiously proposed as an individualized approach, particularly in cases where conventional estrogen therapy is not suitable. While further research is needed, these preliminary data support the integration of PRP into a broader framework of personalized menopausal care.

Future research should focus on long-term, multicenter randomized controlled trials that include larger and more diverse populations. Objective biological assessments, such as epithelial thickness, vascularization, and hormonal receptor activity, could help clarify the mechanisms of PRP and support its role as a hormone-free alternative in postmenopausal sexual health.

## 5. Conclusions

Intravaginal PRP therapy represents a promising non-hormonal alternative for improving sexual function and vaginal health in postmenopausal women. Despite the absence of statistically significant differences compared to local estrogen treatment, its clinical benefits, high patient satisfaction, and favorable safety profile position PRP as a viable therapeutic option, especially for women with contraindications to hormone use.

The observed trends in older patients and those many years postmenopause support its potential in subgroups that are less responsive to estrogen. Future studies with longer follow-up periods and objective biological endpoints are warranted to validate and expand these findings.

## Figures and Tables

**Table 1:** Baseline characteristics of study participants.

Characteristic	PRPG (*n* = 45)	HG (*n* = 45)	*p* Value
Age (years) *	57.2 ± 4.3	56.8 ± 4.7	0.68
Years since menopause *	6.1 ± 2.7	6.3 ± 2.5	0.74
BMI (kg/m^2^) *	26.4 ± 3.1	25.9 ± 3.4	0.58
Married/cohabiting **	38 (84.44%)	36 (80.00%)	0.59
Smoking status (current) **	6 (13.33%)	7 (15.55%)	0.76
FSFI total score *	18.2 ± 3.5	18.6 ± 3.2	0.61
VHI score *	12.3 ± 2.8	12.5 ± 3.1	0.78
Prior local hormonal therapy **	4 (8.88%)	5 (11.11%)	0.72

* mean ± SD, ** percentage.

**Table 2:** FSFI domain scores.

Domain	PRPG	PRPG 95% CI	HG	HG 95% CI	*p* Value
Desire	+1.4 ± 0.7	(1.2, 1.6)	+1.3 ± 0.6	(1.12, 1.48)	0.468
Arousal	+1.8 ± 0.8	(1.57, 2.03)	+1.6 ± 0.9	(1.34, 1.86)	0.268
Lubrication	+2.3 ± 0.9	(2.04, 2.56)	+2.1 ± 1.0	(1.81, 2.39)	0.321
Orgasm	+1.6 ± 0.8	(1.37, 1.83)	+1.5 ± 0.7	(1.3, 1.7)	0.529
Satisfaction	+1.7 ± 0.9	(1.44, 1.96)	+1.6 ± 0.9	(1.34, 1.86)	0.599
Pain	−2.9 ± 1.1	(−3.22, −2.58)	−2.7 ± 1.3	(−3.08, −2.32)	0.432

**Table 3:** Secondary outcomes at 12 weeks post-treatment.

Outcome	PRPG	PRPG 95% CI	HG	HG 95%CI	*p* Value
VHI *	+6.2 ± 2.1	(5.59, 6.81)	+5.6 ± 2.3	(4.93, 6.27)	0.199
PGI-I (“Very much” or “Much improved”) **	39 (86.66%)	(76.7%, 96.6%)	37 (82.22%)	(71.1%, 93.4%)	0.772
Patient Satisfaction (Likert scale)					
Very satisfied **	29 (64.44%)	(50.5%, 78.4%)	26 (57.77%)	(43.3%, 72.2%)	0.665
Satisfied **	13 (28.88%)	(15.6%, 42.1%)	14 (31.11%)	(17.6%, 44.6%)	1.00
Neutral or dissatisfied **	3 (6.66%)	(−0.6%, 14.0%)	5 (11.11%)	(1.9%, 20.3%)	0.713
Adverse events, any **	4 (8.88%)	(0.6%, 17.2%)	7 (15.55%)	(5.0%, 26.1%)	0.521
Breast tenderness **	0 (0%)	-	4 (8.88%)	(0.6%, 17.2%)	0.116
Vaginal spotting **	0 (0%)	-	2 (4.44%)	(−1.6%, 10.5%)	0.494
Local transient discomfort **	3 (6.66%)	(−0.6%, 14.0%)	0 (0%)	-	0.241
Treatment discontinuation **	0 (0%)	-	1 (2.22%)	(−2.1%, 6.5%)	1.000

* mean ± SD, ** percentage.

**Table 4:** Adverse events and safety profile.

Adverse Event	PRPG	PRPG 95% CI	HG	HG 95%CI	*p* Value
Any adverse event, *n* (%)	4 (8.88%)	(0.6%, 17.2%)	7 (15.55%)	(5.0%, 26.1%)	0.521
Local transient discomfort (≤48 h)	3 (6.66%)	(−0.6%, 14.0%)	0 (0%)	-	0.241
Mild vaginal burning (≤24 h)	1 (2.22%)	(−2.1%, 6.5%)	0 (0%)	-	1.000
Breast tenderness	0 (0%)	-	4 (8.88%)	(0.6%, 17.2%)	0.116
Vaginal spotting	0 (0%)	-	2 (4.44%)	(−1.6%, 10.5%)	0.494
Treatment discontinuation	0 (0%)	-	1 (2.22%)	(−2.1%, 6.5%)	1.000
Serious adverse events (SAE)	0 (0%)	-	0 (0%)	-	1.000

**Table 5:** Subgroup observations and trends.

Subgroup (*n*)	Outcome	PRP Group	Hormonal Group	Observed Trend
Age ≥ 60 years (*n* = 30)	FSFI Δ Score	+10.4 ± 3.1	+9.2 ± 3.6	PRP higher
	VHI Δ Score	+6.5 ± 2.0	+5.2 ± 2.2	PRP higher
	Pain Domain (FSFI)	–3.0 ± 1.0	–2.4 ± 1.3	PRP higher
Years since menopause > 8 (*n* = 22)	VHI Progression	Continued at week 12	Plateaued after week 6	PRP sustained
	PGI-I “Very Improved”	7/11 (63.6%)	5/11 (45.5%)	PRP higher
BMI ≥ 30 (*n* = 14)	Satisfaction (Likert)	6/7 “satisfied or very satisfied”	4/7	PRP higher

## Data Availability

The data is available from the corresponding author of the study. You may contact the corresponding author for further details and access to the relevant data. Additionally, a copy of the data is also stored in our clinic’s records.

## List of Abbreviations

PRP	Platelet-Rich Plasma
GSM	Genitourinary Syndrome of Menopause
FSFI	Female Sexual Function Index
VHI	Vaginal Health Index
PGI-I	Patient Global Impression of Improvement
CTCAE	Common Terminology Criteria for Adverse Events
HRT	Hormone Replacement Therapy
BMI	Body Mass Index
FSH	Follicle-Stimulating Hormone
ER	Estrogen Receptor
TGF-β	Transforming Growth Factor Beta
PDGF	Platelet-Derived Growth Factor
VEGF	Vascular Endothelial Growth Factor
SAE	Serious Adverse Event
PRPG	Platelet-Rich Plasma Group
HG	Hormonal Group
SD	Standard Deviation
